# Associations of neighborhood social cohesion and changes in BMI—The Maastricht Study

**DOI:** 10.1093/eurpub/ckae109

**Published:** 2024-06-28

**Authors:** Jeffrey A Chan, Annemarie Koster, Jeroen Lakerveld, Miranda T Schram, Marleen van Greevenbroek, Hans Bosma

**Affiliations:** Care and Public Health Research Institute (CAPHRI), Maastricht University, Maastricht, The Netherlands; Department of Social Medicine, Maastricht University, Maastricht, The Netherlands; Department of Physical Medicine and Rehabilitation, Northern California VA Healthcare System, Martinez, CA, United States; Care and Public Health Research Institute (CAPHRI), Maastricht University, Maastricht, The Netherlands; Department of Social Medicine, Maastricht University, Maastricht, The Netherlands; Department of Epidemiology and Data Science, Amsterdam UMC, Vrije Universiteit Amsterdam, Amsterdam, The Netherlands; Cardiovascular Research Institute Maastricht (CARIM), Maastricht University, Maastricht, The Netherlands; Department of Internal Medicine, Maastricht University, Maastricht, The Netherlands; Heart and Vascular Centre, Maastricht University Medical Centre, Maastricht, The Netherlands; Cardiovascular Research Institute Maastricht (CARIM), Maastricht University, Maastricht, The Netherlands; Department of Internal Medicine, Maastricht University, Maastricht, The Netherlands; Care and Public Health Research Institute (CAPHRI), Maastricht University, Maastricht, The Netherlands; Department of Social Medicine, Maastricht University, Maastricht, The Netherlands

## Abstract

The role of the social environment can facilitate positive health outcomes through active community engagement, normalization of healthy behaviors, and stress buffering. We aim to examine the associations of neighborhood social cohesion with changes in BMI over time. A total of 7641 participants from The Maastricht Study between the ages of 40 and 75 years were analyzed. Weight and height were measured at baseline, and weight was self-reported annually up to 10 years of follow-up (median = 4.7 years). Perceived social cohesion was obtained by questionnaire. Home addresses for each participant were linked to geographic information system data from the Geoscience and Health Cohort Consortium to create neighborhood exposure variables including area level social cohesion, neighborhood walkability, and food environment within a 1000 m Euclidian buffer. Linear regression analyses were performed with BMI adjusted for socioeconomic variables. A mixed model analysis was carried out to examine changes in BMI. Living in the highest quartile area of individually perceived social cohesion was associated with lower BMI (Q4 B: −.53; 95% CI = −.79, −.28) compared to the lowest quartile. Similar findings were discovered using the area level measure (Q4 B: −.97; 95% CI = −1.29, −.65). There was no longitudinal association between social cohesion and BMI. Neighborhood social cohesion was associated with lower BMI classifying it as an obesogenic area characteristic that influences weight, independent of conventional built environment features.

Key points

- BMI can be influenced by the social environment independent of walkability and food environment.
- The social environment should be observed as an upstream risk factor for high BMI.
- Neighborhood administrative data may serve as a proxy for social cohesion.
- Clinicians should be aware that patients may face additional challenges based on their residential location.

## Introduction

The link between obesity and cardiovascular disease as well as other chronic conditions has been well established [1, 2]. Genetic predisposition aside, obesity is influenced and managed by lifestyle behaviors (e.g. diet, exercise), and public health interventions have traditionally focused on campaigns to address these behaviors [3]. In the past two decades, research has evaluated upstream features that contribute to obesity such as the food environment and neighborhood walkability that are key aspects of the obesogenic environment [4]. The conceptual theory is that more walkable and healthier food environments drive healthier food consumption and increased physical activity. Thus far, reviews on the association between walkability and food environment with measures of weight status have been inconclusive with some studies observing findings contrary to what has been expected [5, 6].

Branching out further to non-conventional approaches to address the rise in obesity, attention has turned to evaluating the social environment as an obesity determinant where individuals are inveigled by their peers [7]. *Social cohesion* particularly is a concept characterized by residents who are engaged, trusting, and respectful that can influence one another through their decisions and actions [8, 9]. At the neighborhood level, these relationships can offer social support and encourage individuals to access neighborhood resources they may have otherwise not known or utilized [10]. On the other hand, residents in areas of low social cohesion have high levels of stress, which is linked to chronic elevated cortisol levels that can increase appetite and eventually lead to weight gain [11, 12]. This is problematic for those living in lower income neighborhoods who are more prone to overeating and other detrimental coping responses to address stressful situations [11, 13]. Alternatively, when individuals feel safe and trust others around them, they are more likely to practice favorable behaviors such as spending time outdoors and taking part in regular physical activity [14]. Greater social cohesion can result in positive dietary behaviors that can be modeled and observed [15]. It is possible that the social environment can promote favorable lifestyle behaviors when ideal food environments and walkable neighborhoods are not immediately accessible.

Social cohesion has been primarily measured through a questionnaire that attempts to quantify an individual’s perception of their neighborhood [16, 17]. The information obtained conveys resident experiences with social relationships and trust with those amongst them that are necessary to access resources and buffer stress. Furthermore, a recently developed instrument by the Dutch Ministry aims to capture area correlates of social cohesion [18]. This validated scale intends to utilize administrative neighborhood measures that may facilitate a cohesive environment. These two indices simultaneously allow for examining perceived and contextual mechanisms of how social cohesion may cast its influence physiologically and behaviorally.

Thus far, several papers have examined the relationship between social cohesion and obesity, independent of other built environment features, with mixed results. Early findings have established that such relationship exists but differs by various populations and covariates [19]. Some of these conditions are influenced by sex, sample size, cultures, heterogeneity, and location. Almost all research on this topic has been cross-sectional and entirely reliant on self-reported weight. In addition, most of the research has been carried out in North America. To the best of our knowledge, there are no studies that examine the social and obesogenic environment in relation to obesity outcomes longitudinally.

The aim of this paper is to examine the cross-sectional and longitudinal associations of neighborhood social cohesion and BMI across up to 10 years of follow-up. Moreover, we aim to understand if the association is independent of neighborhood walkability and food environment. We will utilize individually perceived social cohesion as well as an area level proxy of social cohesion. We hypothesize that greater social cohesion, individually perceived and area measured, is associated with lower BMI, independent of neighborhood walkability and food environment.

## Methods

### Study population

Data were used from The Maastricht Study, an observational prospective cohort study. The study is set in a fairly homogenous, moderately sized city including surrounding suburban areas. The rationale and methodology have been described previously [20]. In brief, the study focuses on the etiology, pathophysiology, complications, and comorbidities of type 2 diabetes mellitus (T2DM) and is characterized by an extensive phenotyping approach.

Eligibility for participation was individuals between the ages of 40 and 75 years and living in the southern part of The Netherlands. Participants with and without diabetes were recruited through mass media campaigns, from the municipal registries, and the regional Diabetes Patient Registry via mailings. Recruitment was stratified according to known T2DM status, with an oversampling of individuals with T2DM for reasons of efficiency. The present report includes cross-sectional data from the first 9188 participants, who completed the baseline survey between November 2010 and October 2020. The examinations of each participant were performed within a time window of 3 months.

### BMI

Height (measured in centimeters) and weight (kilograms) were objectively measured at study baseline by a trained technician. BMI was calculated using the standard formula kg/m^2^. Self-reported weight (kg) was collected from annual follow-up questionnaires up to a period of 10 years; baseline height was used in the follow-up BMI calculation. BMI ranges are as follows: normal weight (18.5–24.9), overweight (25.0–29.9), and obesity (≥30.0). Those with implausible values, as defined as greater than 3 standard deviation changes in weight from the previous year during each follow-up, were excluded (*n* = 54).

### Social cohesion

Perceived social cohesion was gathered from participant completion of the Dutch-derived Social Cohesion and Trust scale [16] at baseline for The Maastricht Study. This 5-item scale includes the following statements: “People around here are willing to help their neighbors”; “People in my neighborhood share the same values”; “This is a close-knit neighborhood”; “People in my neighborhood can be trusted”; “People in my neighborhood do not share the same values.” The scale employs five Likert response options ranging from “strongly disagree” to “strongly agree,” with possible scores ranging from 5 to 25 (higher scores indicating greater social cohesion). Sum scoring of the questionnaire was used as each item of the scale was equally weighted as with previous work [17]. In the present study, the scale had good internal consistency (Cronbach’s α = .83). This scale was operationalized as an individual measure given our aim to understand participant perceptions serving as a proxy for such mechanisms as social support to buffer stress. Previous studies have aggregated this scale though we believed it might be more conducive to use individual scores given our specific research question accounting for real world perception along with self-reported weight on annual follow-up.

Geographic information systems (GIS) data were obtained from the Geoscience and Health Cohort Consortium (GECCO). In GECCO, environmental variables are centralized, operationalized into personal environmental exposures, and used to enrich cohort studies by being linked to individual-level health data upon request from researchers [21, 22]. GECCO data were linked to participant home address at the year of entry for The Maastricht Study.

Area-measured social cohesion was obtained from the Livability Meter 3.0 and operationalized as an estimate of the social conditions that may have driven cohesiveness and ensuing health behaviors. The Livability Meter was developed by the Dutch Ministry of the Interior and Kingdom Relations, an instrument that—using five dimensions derived from the Housing Survey in The Netherlands—estimates quality of life at a scale of 100 × 100-meter grids, which participants’ home addresses were matched to [23]. The instrument aims to be a monitoring tool to predict the opinions of residents about their neighborhood. This measure, depending on region, explains 50%–74% of the variance in participant survey responses using the perceived social cohesion scale as reference [18]. Social cohesion is one of the five components of livability and was formulated by (1) Diversity by Life Stage, (2) Population Density, and (3) Mutation Rate. Diversity of the population by life stage refers to demographic variation within a neighborhood. This includes age groups that span from infanthood to young adult, middle-aged adults, and older adults. Additionally, life stage diversity differentiates students, single vs two person households, and families with young or older children. On average, the most diverse the neighborhood environment is, the more “difficult” it is for its residents to create a social environment. It is believed that this may be due to the confluence of individuals with conflicting lifestyle needs. As a result, the frequency of mutual contacts is less along with lower participation in the local community [24, 25]. Population density refers to the number of residents within a defined geographical area. With the highest levels, it has been shown to have a negative relationship with the social environment: As the number of residents increases, familiarity with one another decreases. Mutation rate is a measure of residential turnover for a specific area over a period of time. Like population density, the less familiarity with individuals in the community leads to fewer interactions and trust among residents as they actively need to rebuild new relationships with one another with higher mutation rate. Those who remain permanent in a neighborhood amongst high levels of transience are more prone to isolate. Further details can be found in the instrument development document [18].

### Covariates

Covariates in the main analysis included age, sex, type 2 diabetes (T2DM) status (due to oversampling), and education. Presence of T2DM was measured by a glucose tolerance test and medication use [20]. Education was divided into low (no education, primary education, and lower vocational education); medium (general secondary education, general vocational education, and higher secondary and pre-university education); or high (higher vocational education and university). Covariates used in the sensitivity analyses were property value, household income, and occupational status. Neighborhood property value was obtained from Statistics Netherlands and provided by GECCO. Property value was calculated using the mean postcode property value in Euros across the data collection period from 2010 to 2019. Household income was measured in The Maastricht Study by self-reported net household income per month, and the equivalent income was computed by household income divided by the square root of household size [26]. Occupational status was classified under the International Socio-Economic Index of Occupational Status (ISEI-08).

The neighborhood walkability from GECCO was operationalized as an index, calculated based on a combination of seven components (e.g. street connectivity, greenspace) within a 1000 m Euclidian buffer zone. The index was scaled 0–100 with the most walkable neighborhoods having the higher value. This objective scale is described in detail elsewhere [27].

Perceived walkability was obtained in The Maastricht Study from the Abbreviated Neighborhood Environment Walkability Scale (ANEWS) that was gathered at baseline for the Maastricht Study. This validated instrument included statements about the neighborhood in relation to walkability across eight domains [28]. Further details can be found elsewhere [29].

Data on the food environment obtained from GECCO were based on the LOCATUS database consisting of a comprehensive audit taken every 2–3 years of available food outlets in the study region that included markets and restaurants within a 1000 m Euclidian buffer zone from each participant home address. Food establishments were scored −5 to +5 with healthier stores rated a positive and higher score while those that offered unhealthy foods were assigned a lower, negative score. Details on the food environment can be found in previous studies [30].

### Statistical analysis

Baseline characteristics were investigated, and data were summarized using percentages (categorical variables). Multivariate linear models were performed with unstandardized BMI (from the initial measure) as the outcome. The exposure variables include perceived and Livability Meter-measured social cohesion in separate models. To estimate the association of social cohesion and BMI change, we utilized mixed model analyses to model the repeated measures of BMI during follow-up in relation to the fixed effects of social cohesion at baseline.

Model 1 was the unadjusted model. Covariates included age, sex, and attained education level in Model 2; and T2DM (due to oversampling) was added to Model 3. The fully adjusted model included the addition of neighborhood walkability and food environment combined in Model 4.

Sensitivity analyses performed included multivariate linear regression using obesogenic environment exposure variables of objective walkability, perceived walkability, and food environment. Effect modification by sex, age, and socioeconomic position was tested by interactions with age, sex, T2DM, education, income, and housing value. To further exclude socioeconomic confounding, models were adjusted for income, property value, and occupational status instead of attained education.

## Results

Participants were excluded for missing data on perceived social cohesion *n* = 990, missing data on education = 316, address could not be matched to GIS data (or moved), or consent was not given *n* = 241, leaving a sample size of *n* = 7641. Sample characteristics are presented in Table 1. The mean age of the participants was 59.3, and average BMI was 26.7. Men had a higher BMI (27.3) than women (26.2), and the lowest education group had a higher BMI (27.9) than the highest education group (25.7). Those living in neighborhoods with the lowest quartile of social cohesion, walkability, and food environment had a higher average BMI than participants in the highest quartile.

**Table 1.** Population characteristics by mean body mass index

Age	
59.3 (mean avg)	26.7
Sex	
Male (49.4%)	27.3
Female (50.6%)	26.2
Education level	
Low	27.9
Medium	26.8
High	25.7
Diabetes type 2	
No	26.0
Yes	29.8
Perceived social cohesion	
Q1 (Lowest)	27.3
Q2	26.8
Q3	26.2
Q4 (Highest)	26.5
Livability Meter social cohesion	
Q1	27.3
Q2	26.8
Q3	26.5
Q4	26.3
Perceived walkability	
Q1	27.2
Q2	27.0
Q3	26.6
Q4	26.1
Objective walkability	
Q1	26.8
Q2	27.0
Q3	26.9
Q4	26.3
Food environment	
Q1	27.1
Q2	26.4
Q3	26.8
Q4	26.7

Table 2 shows the cross-sectional associations between social cohesion and BMI. In Model 3, those living in the highest quartile area of perceived social cohesion had significantly lower BMI compared to the lowest quartile (Q4 B: −.50; 95% CI = −.78, −.22). Similar findings using the Livability Meter measure of social cohesion were found in the highest quartile for social cohesion (Q4 B: −.50; 95% CI = −.79, −.22). After further adjustments for neighborhood walkability and food environment (Model 4), both perceived (Q4 B: −.53; 95% CI = −.79, −.28) and Livability Meter-measured (Q4 B: −.97; 95% CI= −1.29, −.65) remained significant.

**Table 2.** Cross-sectional association of social cohesion and BMI *B* (95% CI)

	Model 1	Model 2	Model 3	Model 4
Perceived social cohesion				
Q1	Ref	Ref	Ref	Ref
Q2	−.47 (−.77, −.17)*	−.36 (−.66, −.07)*	−.21 (−.49, .07)	−.19 (−.44, .07)
Q3	−1.03 (−1.35, −.71)*	−.55 (−.85, −.25)*	−.55 (−.85, −.24)*	−.61 (−.89, −.33)*
Q4	−.77 (−1.07, −.47)*	−.50 (−.78, −.22)*	−.50 (−.78, −.22)*	−.53 (−.79, −.28)*
Livability Meter social cohesion				
Q1	Ref	Ref	Ref	Ref
Q2	−.42 (−.72, −.12)*	−.22 (−.52, .07)	−.13 (−.42, .15)	−.13 (−.39, .13)
Q3	−.69 (−1.00, −.39)*	−.44 (−.74, −.14)*	−.28 (−.57, .00)	−.52 (−.80, −.24)*
Q4	−.91 (−1.22, −.61)*	−.65 (−.95, −.35)*	−.50 (−.79, −.22)*	−.97 (−1.29, −.65)*

Quartile 1 is the lowest; Quartile 4 is the highest. Model 1: Crude. Model 2: Adjusted for age, sex, education. Model 3: Adjusted for Model 2 + type 2 diabetes. Model 4: Adjusted for Model 3 + obesogenic environment (walkability and food environment).

* *P* < .05.

**Figure 1.**
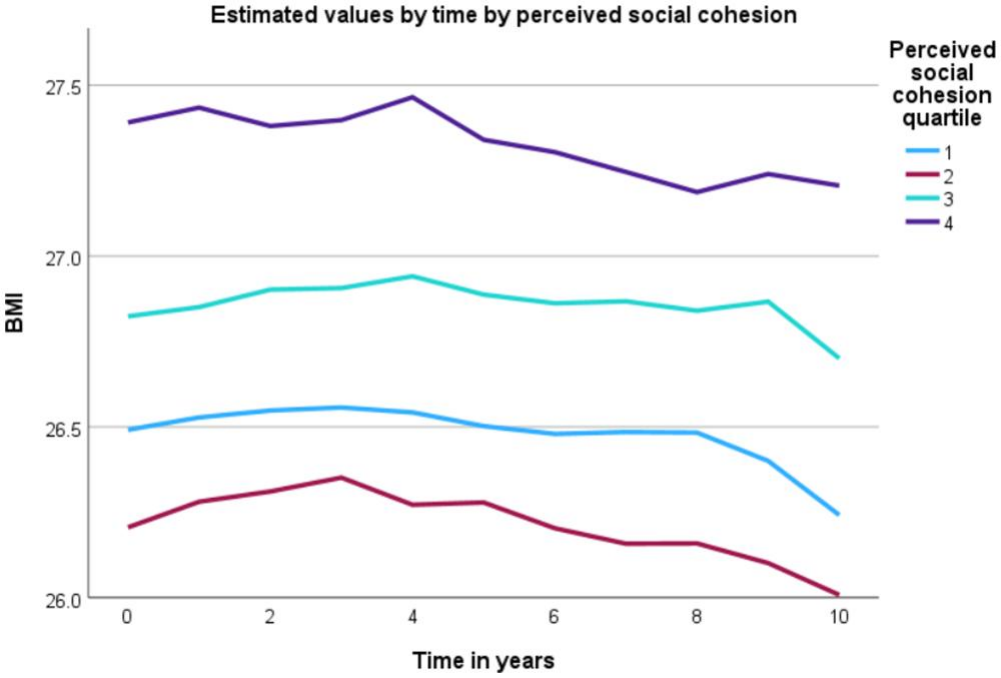
Mean BMI value by perceived social cohesion quartile across up to a 10-year follow-up period.

Table 3 yields longitudinal findings of the social environment and BMI. There was no statistically significant cohesion × time interaction (median = 4.7 years follow-up) for participants in the highest quartile in both perceived (B: −.11; 95% CI = −.38, .15) and Livability Meter-measured (B: −.14; 95% CI = −.42, .15) social cohesion (Fig. 1, Supplementary Fig. S1).

**Table 3.** Social cohesion exposure and longitudinal changes in BMI *B* (95% CI)

	Model 1	Model 2	Model 3	Model 4
Perceived social cohesion	Intercept	Intercept	Intercept	Intercept
Q1	Ref	Ref	Ref	Ref
Q2	−.58 (−.82, −.34)*	−.38 (−.62, −.15)*	−.16 (−.62, −.15)*	−.16 (−.38, .07)
Q3	−1.20 (−1.47, −.93)*	−.81 (−1.08, −.54)*	−.56 (−1.08, −.54)*	−.57 (−.82, −.31)*
Q4	−.91 (−1.16, −.67)*	−.67 (−.91, −.44)*	−.48 (−.91, −.44)*	−.49 (−.72, −.26)*
	Time interaction	Time interaction	Time interaction	Time interaction
Q1	Ref	Ref	Ref	Ref
Q2	.05 (−.22, .32)	.04 (−.25, .29)	.02 (−.23, .31)	.02 (−.25, .29)
Q3	−.08 (−.37, .21)	−.09 (−.40, .18)	−.10 (−.38, .21)	−.11 (−.40, .18)
Q4	−.09 (−.36, .18)	−.10 (−.38, .16)	−.11 (−.37, .18)	−.11 (−.38, .15)
Livability Meter social cohesion	Intercept	Intercept	Intercept	Intercept
Q1	Ref	Ref	Ref	Ref
Q2	−.57 (−.82, −.32)*	−.31 (−.56, −.07)*	−.12 (−.35, .11)	−.09 (−.32, .15)
Q3	−.85 (−1.10, −.60)*	−.52 (−.76, −.27)*	−.28 (−.51, −.04)*	−.41 (−.66, −.16)*
Q4	−1.06 (−1.31, −.81)*	−.74 (−.98, −.49)*	−.50 (−.73, −.26)*	−.79 (−1.06, −.51)*
	Time interaction	Time interaction	Time interaction	Time interaction
Q1	Ref	Ref	Ref	Ref
Q2	−.03 (−.30, .24)	−.04 (−.31, .23)	−.05 (−.32, .21)	−.06 (−.33, .21)
Q3	−.10 (−.37, .17)	−.11 (−.37, .16)	−.12 (−.39, .15)	−.13 (−.39, .14)
Q4	−.11 (−.40, .18)	−.12 (−.40, .17)	−.13 (−.42, .16)	−.14 (−.42, .15)

Quartile 1 is the lowest; Quartile 4 is the highest. Model 1: Crude. Model 2: Adjusted for age, sex, education. Model 3: Adjusted for Model 2 + type 2 diabetes. Model 4: Adjusted for Model 3 + obesogenic environment (walkability and food environment).

* *P* < .05.

Sensitivity analyses examining the obesogenic environment and BMI were performed. Supplementary Table S1 displays the full model controlling for social cohesion was statistically significant in both perceived (Q4 B: −.75; 95% CI = −1.02, −.49) and Livability Meter-measured neighborhood walkability models (Q4: −1.07; 95% CI = −1.43, −.72). There was no association with the food environment controlling for social cohesion (Q4 B: −.06; 95% CI = −.32, .21). Supplementary Table S2 reveals longitudinal analyses did not find statistically significant time interaction using neighborhood walkability (B: −.02; 95% CI = −.04, .00). The food environment exhibited a statistically significant decrease in BMI (B: −.02; 95% CI = −.04, 4.0 e ^−3^).

Effect modification analyses produced a statistically significant interaction towards lower BMI with the lowest attained education group using the Livability Meter social cohesion scale but not using the perceived social cohesion questionnaire. There were otherwise no other significant interactions regarding demographic and socioeconomic status variables (untabulated). The full linear model adjusted for income, property value, and then occupational status instead of attained education yielded similar results as the main analyses (Supplementary Table S3).

## Discussion

This study investigated the relationship between the social environment and changes in BMI. We show that higher individually perceived and area-measured social cohesion were associated with a lower BMI independent of the obesogenic environment. There was no statistically significant longitudinal association between social cohesion and BMI as consistent differences in BMI between social cohesion groups persisted, but these differences did not diverge over time.

Our findings show that greater neighborhood social cohesion is associated with lower BMI. As the study of this relationship remains in its infancy, this is one of few papers that has identified this relationship with BMI. A survey of 5199 adults across five European countries discovered neighborhoods with higher social cohesion were associated with lower BMI [31]. In the USA, in an African-American sample, an association was found between higher social cohesion and lower BMI, while no association was shown in a south Asian sample [32, 33].

Our study supports the possibility that relationships and social resources forged by higher social cohesion are associated with lower BMI [8, 34]. Some of the additional pathways proposed by Kawachi and Berkman [35] explaining the effect of social cohesion on health behaviors include the diffusion of new health information, reduced occurrences of social isolation, and civic engagement. More cohesive neighborhoods are likely to be aware of and assist those in distress while bringing attention to existing resources [36]. Additionally, members of these neighborhoods are more likely to unite and lobby for social services and facilities to address their needs through community organization [37]. Thus, it appears a confluence of these factors may have contributed to a physiological state, as well as participation in behaviors, that appears to have been complementary in maintaining a stable energy balance. Further research should study neighborhoods with high social cohesion and identify specific behaviors, such as physical activity and dietary quality, that may lead to lower BMI.

This is one of the first studies to use a perceived and area measure of social cohesion. Though the Social Cohesion and Trust scale and its variants have been commonly utilized in the literature, its use is not without limitations [17]. The self-report measure is vulnerable to reverse causation bias, and participants may misattribute their circumstances to their environment [38]. Given the single administration of this measure without follow-up, we are not able to be definitive of the causal direction. Moreover, the interpretation of the Social Cohesion and Trust scale was developed in North America, and statements included in the measure can be misinterpreted by other cultures [18]. However, we used a Dutch-derived version of this scale as well as incorporated an area measure (Livability Meter) developed specifically for the Dutch region to increase validity. Furthermore, the area Livability Meter measure uses contextual neighborhood characteristics associated with social cohesion based on population surveys though not a direct measure of social cohesion itself. It has not yet been established if the subcomponents of the Livability Meter are resultant or causal of neighborhood social cohesion. However, the use of both scales measuring different mechanisms of the social environment yielded similar results which corroborates our findings.

We did not observe statistically significant changes in BMI during the follow-up period. Our demographic comprised primarily of middle-aged and older participants. Given this information, the effect of social cohesion may not have been enough to counteract weight fluctuation that is a natural part of the aging process in this demographic as a result of metabolic changes [39]. However, our findings show that differences remained between the quartiles including those in the lowest quartile of social cohesion. We presume that behaviors already developed from existing residency may have already been established before data collection. Further studies should be carried out amongst younger adults where weight change may be more sensitive to the social and obesogenic environment.

We examined built environment exposures traditionally associated with BMI in our sensitivity analyses which included walkability and the food environment. There was an association with the highest walkable neighborhood using the perceived scale but not the objective measure or food environment with BMI. These findings reinforce that the social environment is associated with lower BMI independent of neighborhood walkability and food environment.

### Strengths and limitations

There are several strengths to this study. First, we studied a large sample size in addition to participants completing follow-up measures allowing us to adjust for multiple socioeconomic confounders. Second, this is one of the first studies to utilize an area level measure of social cohesion using neighborhood correlates, alongside a subjective questionnaire, examining the different mechanisms of how the social environment may serve to influence behaviors leading to lower BMI. Third, this was one of few studies to examine the longitudinal association between the social environment and BMI. Finally, we were able to adjust for the possibility of confounding by neighborhood walkability and food environment.

One of our limitations is that although our study found an association between social cohesion and BMI, we are unable to infer a causal relationship. Further studies such as mediation and additional longitudinal studies should be conducted in continuing to understand this pathway. Second, participant-reported weights were collected on annual surveys to measure longitudinal BMI change. Self-reported weight is prone to desirability bias resulting in underestimation of weight. Despite this shortcoming, the accuracy of self-reported weight has recently been shown to be valid [40]. We were also able to establish a baseline weight for each participant at the study center which did not deviate much from the first self-reported weight resulting in fairly accurate self-reporting. Finally, we found a large number of individuals with missing data on the perceived cohesion questionnaire. Those individuals were discovered to be proportionally similar in BMI, age, sex, and socioeconomic characteristics to the sample we analyzed.

Though not directly observable, the social environment plays a role in the physiological state and an influential presence in the BMI of individuals. We found neighborhoods with higher social cohesion were associated with lower BMI independent of walkability and the food environment. Clinicians should factor in the residential location and the accompanying challenges certain patients may face when developing weight management programs.

## Supplementary Material

ckae109_Supplementary_Data

## Data Availability

The data underlying this article cannot be shared publicly due to privacy of individuals that participated in the study. The data will be shared on reasonable request to the corresponding author.
