# Correction to: Associations of neighborhood social cohesion and changes in BMI—The Maastricht Study

**DOI:** 10.1093/eurpub/ckae170

**Published:** 2024-10-29

This is a correction to: Jeffrey A Chan, Annemarie Koster, Jeroen Lakerveld, Miranda T Schram, Marleen van Greevenbroek, Hans Bosma, Associations of neighborhood social cohesion and changes in BMI—The Maastricht Study, *European Journal of Public Health*, Volume 34, Issue 5, October 2024, Pages 949–954, https://doi.org/10.1093/eurpub/ckae109

The following change has been made to the originally published article. The text in the caption for Figure 1 has been emended to read: “Figure 1. Mean BMI value by **perceived **social cohesion quartile across up to a 10-year follow-up period” instead of: “Figure 1. Mean BMI value by **Livability Meter-measured** social cohesion quartile across up to a 10-year follow-up period”.

The emendation has been made to the article.

