# Supplementary material for: Associations of neighborhood social cohesion and changes in BMI—The Maastricht Study
Source: Eur J Public Health. 2024 Jun 28;34(5):949–54. doi: 10.1093/eurpub/ckae109 (PMC11430969; doi:10.1093/eurpub/ckae109)
Supplement: ckae109_Supplementary_Data [file ckae109_supplementary_data.zip › ckae109_Supplementary_Data/ejph-2024-01-om-0026-File003.pdf]

Supplementary Table 1: Association of obesogenic environment and BMI *B (95% CI)*

	Model 1	Model 2	Model 3	Model 4
**Objective** Walkability				
Q1	Ref	Ref	Ref	Ref
Q2	.32 (.01, .62)*	.26 (-.04, .55)	.30 (.01, .58)*	.25 (-.54, .04)
Q3	.20 (-.11, .51)	.14 (-.16, .44)	.17 (-.12, .46)	-.65 (-.97, .33)
Q4	-.32 (-.63, -.02)*	-.18 (-.48, .12)	-.09 (-.38, .19)	-1.07 (-1.43, -.72)*
**Perceived** Walkability				
Q1	Ref	Ref	Ref	Ref
Q2	-.17 (-.49, .15)	-.19 (-.50, .19)	-.16 (-.45, .14)	-.25 (-.51, .02)
Q3	-.46 (-.75, -.17)*	-.34 (-.63, -.06)*	-.23 (-.51, .04)	-.39 (-.64, -.14)*
Q4	-.97 (-1.27, -.67)*	-.76 (-1.05, -.46)*	-.59 (-.87, -.31)*	-.75 (-1.02, -.49)*
Food Environment				
Q1	Ref	Ref	Ref	Ref
Q2	-.51 (-.82, -.21)*	-.30 (-.60, -.01)*	-.27 (-.55, .01)	-.47 (-.73, -.22)*
Q3	-.12 (-.43, .19)	.07 (-.23, .38)	.08 (-.21, .37)	-.14 (-.41, .12)
Q4	-.26 (-.58, .05)	-.01 (-.32, .30)	-.00 (-.30, .29)	-.06 (-.32, .21)

**p* value <.05

Quartile 1 is the lowest; Quartile 4 is the highest

Model 1: Crude

Model 2: Adjusted for Age, Sex, Education

Model 3: Adjusted for Model 2 + Type 2 Diabetes

Model 4: Adjusted for Model 3 + Livability Meter Social Cohesion**

**The use of the Livability Meter and perceived social cohesion separately yielded similar findings in the model

Supplementary Table 2: Mixed models time interaction of obesogenic environment exposure with BMI

	B (95% CI)	
	Intercept	Time Interaction
**Objective Neighborhood Walkability**		
Q1	Ref	Ref
Q2	.09 (-.14, .34)	.01 (-.01, .03)
Q3	-.01 (-.25, .23)	-.02 (-.00, .04)
Q4	-.28 (-.52, -.04)*	.02 (-.00; .04)
**Perceived Neighborhood Walkability**		
Q1	Ref	Ref
Q2	-.09 (-.33, .16)	.01 (-.01, .03)
Q3	-.21 (-.44, .01)	.02 (-.00, .03)
Q4	-.56 (-.80, -.32)*	.01 (-.01, .03)
**Food Environment**		
Q1	Ref	Ref
Q2	-.40 (-.64, .18)*	-.00 (-.02, .02)
Q3	-.04 (-.28, .20)	.-01 (-.02, .01)
Q4	-.06 (-.31, .18)	-.02 (-.04, 4.0 e-3)*

*p value <.05

Quartile 1 is the lowest; Quartile 4 is the highest

Adjusted for Age, Sex, Education, Type 2 Diabetes, Livability Meter Social cohesion**

**The use of the Livability Meter and perceived social cohesion separately yielded similar findings in the model

Supplementary Table 3: Associations of social and built environment and BMI (Education substituted for alternate socioeconomic variables) B (95% CI)

	Income	Property Value	Occupational Classification
**Perceived** Social Cohesion			
Q1	Ref	Ref	Ref
Q2	-.25 (-.54, .03)	-.28 (-.53, -.02)*	.02 (-.36, .41)
Q3	-.64 (-.95, -.33)*	-.82 (-1.10. -.55)*	-.41 (-.83, .00)
Q4	-.53, (-.81, -.24)*	-.63 (-.89, -.37)*	-.24 (-.64, .16)
**Livability Meter**			
Social Cohesion			
Q1	Ref	Ref	Ref
Q2	-.19 (-.47, .10)	-.25 (-.51, .01)	-.08 (-.46, .30)
Q3	-.35 (-.64, -.06)*	-.46 (-.72, -.20)*	-.34 (-.74, .06)
Q4	-.56 (-.85, -.27)*	-.69 (-.95, -.43)*	-.69 (-1.11, -.28)*
**Perceived** Walkability			
Q1	Ref	Ref	Ref
Q2	-.13 (-.42, .17)	-.13 (-.40, .14)	-.27 (-.68, .14)
Q3	-.25 (-.53, .02)	-.38 (-.63, -.13)*	-.55 (-.94, -.17)*
Q4	-.65 (-.93, -.36)*	-.80 (-1.06, -.54)*	-.57 (-.97, -.17)*
**Objective** Walkability			
Q1	Ref	Ref	Ref
Q2	.31 (.03, .60)*	.27 (.00, .53)*	.42 (-.01, .84)
Q3	.18 (-.11, .47)	.14 (-.13, .40)	.42 (.01, .83)*
Q4	-.17 (-.45, .12)	-.28 (-.54, -.02)*	-.21 (-.63, .20)
Food Environment			
Q1	Ref	Ref	Ref
Q2	-.39 (-.67, -.10)*	-.59 (-.85, -.33)*	-.72 (-1.12, -.33)*
Q3	-.00 (-.29, .29)	-.20 (-.47, .06)	-.10 (-.51, .30)
Q4	-.09 (-.38, .21)	-.28 (-.55, -.01)*	-.34 (-.75, .10)

**P*-Value <.05
Quartile 1 is the lowest; Quartile 4 is the highest
Income Model: Adjusted for Age, Sex, Diabetes, Income
Property Value Model: Adjusted for Age, Sex, Diabetes, Property Value
Occupational Classification Model: Adjusted for Age, Sex, Diabetes, ISEI08 (Occupational Classification)
