# Supplementary material for: Associations of neighborhood social cohesion and changes in BMI—The Maastricht Study
Source: Eur J Public Health. 2024 Jun 28;34(5):949–54. doi: 10.1093/eurpub/ckae109 (PMC11430969; doi:10.1093/eurpub/ckae109)
Supplement: ckae109_Supplementary_Data [file ckae109_supplementary_data.zip › ckae109_Supplementary_Data/ejph-2024-01-om-0026-File004.pdf]

Appendix A – Obesogenic Environment: Walkability and Food Environment

**Operationalization of the food environment index:**

Using the types of food outlets from LOCATUS, a Delphi study conducted by an expert panel was performed to create an aggregate measure of the quality of the retail establishments in the Netherlands, generating the Food Environment Healthiness Index (FEHI). The FEHI index classifies each type of food retailer in relation to the nutritional quality of the foods they offer on a scale from –5.0 for the least healthy to +5.0 for the healthiest. Thus, a positive score is considered healthy and a negative score is less healthy. For example, a fish retailer and a supermarket were given a score of 2.8 and 1.8, respectively. A liquor store and confectionary shop were assigned values of –4.6 and –4.7. All scores of all food outlets averaged to zero in a kernel around the outlets, creating a heatmap with assigned FEHI scores for the full region (Fig. 1). An analysis was conducted evaluating a participant's broader food environment using the FEHI that was assigned to their address area. In addition, the density of the six most common types of food outlets frequented was individually evaluated and included in the main analysis: local food shops (LF) including a butcher shop and bakery, fast food (FF), food delivery (FD), restaurants (RS), supermarkets (SM) and convenience stores (CS). The individual count of each of these establishments was calculated for each participant's address. A Euclidian buffer zone of 1000 m was used as a reference distance from an individual address in relation to nearby food outlets. This allowed each participant to have their own residential food environment analyzed. The distance of 1000 m was chosen in the main analysis as it is the best estimate within walking and cycling range for the average individual to travel to each food outlet, accounting for suburban neighborhoods.

Obtained from: Chan JA, Koster A, Eussen SJ, et al. The association between the food environment and adherence to healthy diet quality: the Maastricht Study. Public Health Nutr. 2023;26(9):1775-1783. doi:10.1017/S1368980023001180

**Operationalization of the objective neighborhood walkability index:**

The neighborhood walkability measures were calculated based on a combination of selected components. The components were the following environmental characteristics with the higher score indicating more walkable: (1) population density: The number of inhabitants per square kilometer; (2) density of retail and service destinations: The proportion of area retail, hospitality, social services and hospitality; (3) land-use mix: Area devoted to residential, commercial, social cultural, public service and office space, and greenspace; (4) street connectivity: Density of ≥3-way intersections; (5) green space: Area devoted to parks, forests, and cemeteries; (6) sidewalk density: Proportion of sidewalk area; and (7) public transportation density: Public transportation stops accessible on foot or by bike. We chose the walkability data of 2012, 2015, and 2017 as these were closest in time to our enrollment periods (representative of the current neighborhood), contained data from all seven environmental characteristics, as well as when full audits were performed on these subcomponents. Walkability scores of the components retrieved from year 2012 were used for participants entering the Maastricht Study between 2010 and 2013. Walkability scores from 2015 were used for participants enrolled in the Maastricht Study between 2014 and 2016. The remaining participants entering the study in 2017 were assigned to the 2017 enrollment group. The 1000 m Euclidian buffer zone around each home address for each participant was chosen as the default distance for the main analysis.
Altogether, the *z*-scores for buffer zones from the seven components were equally weighted and summed, and the results were rescaled to a value from 0 to 100, with higher scores representing higher walkability levels. Walkability was further ranked into quartiles to facilitate comparisons and allow for possible nonlinear associations. The first quartile (Q1) represented the least walkable neighborhoods and the upper quartiles represented the most walkable neighborhoods: low (Q1), low-to-medium(Q2), medium-to-high (Q3), and high walkable neighborhood (Q4).
Subjective walkability was obtained from the Abbreviated Neighborhood Environment Walkability Scale (ANEWS) that was gathered at baseline for the Maastricht Study. This validated instrument included statements about the neighborhood in relation to walkability across eight domains.The dimensions included were classified into land-use mix (proximity to retail and public destinations), infrastructure (sidewalk maintenance and cycling safety), and traffic (safety from motor vehicles). The responses were adapted into a 5-point scale in the Dutch language as follows: (1) Strongly agree; (2) Agree; (3) Neutral; (4) Disagree; (5) Strongly Disagree. Similar to the objective walkability index, the three components were summed and the results were rescaled to a value from 0 to 100 using similar formula as objective walkability. The ANEWS was also divided into quartiles.

Obtained from: Chan JA, Bosma H, Drosinou C, et al. Association of perceived and objective neighborhood walkability with accelerometer-measured physical activity and sedentary time in the Maastricht Study. Scand J Med Sci Sports. 2023;33(11):2313-2322. doi:10.1111/sms.14455
