# Supplementary figures and images for: Associations of neighborhood social cohesion and changes in BMI—The Maastricht Study
Source: Eur J Public Health. 2024 Jun 28;34(5):949–54. doi: 10.1093/eurpub/ckae109 (PMC11430969; doi:10.1093/eurpub/ckae109)

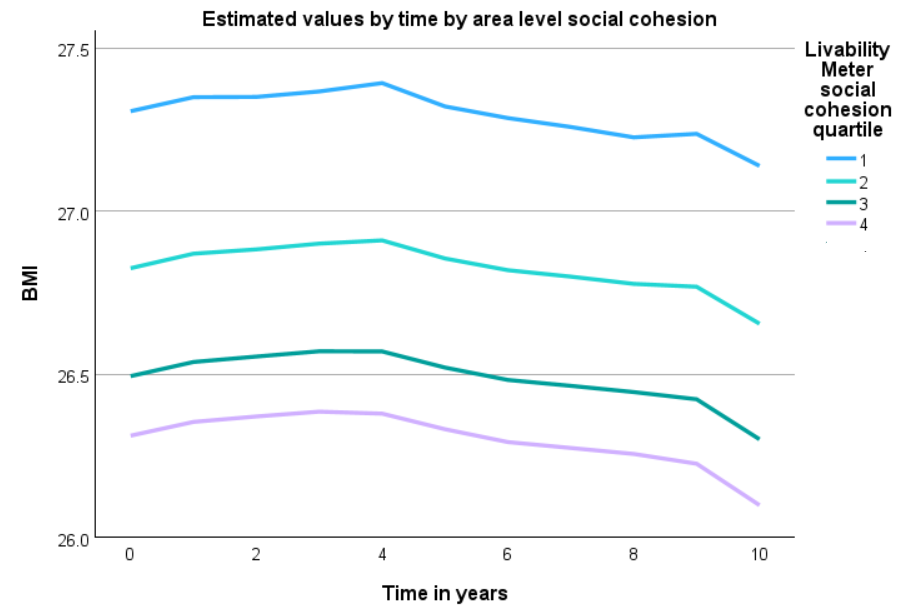

Supplement: ckae109_Supplementary_Data [file ckae109_supplementary_data.zip › ckae109_Supplementary_Data/ejph-2024-01-om-0026-File005.tiff]
